# Crystal structure of 4-chloro-2-nitro­benzoic acid with 4-hy­droxy­quinoline: a disordered structure over two states of 4-chloro-2-nitro­benzoic acid–quinolin-4(1*H*)-one (1/1) and 4-hy­droxy­quinolinium 4-chloro-2-nitro­benzoate

**DOI:** 10.1107/S205698901901497X

**Published:** 2019-11-08

**Authors:** Kazuma Gotoh, Hiroyuki Ishida

**Affiliations:** a Department of Chemistry, Faculty of Science, Okayama University, Okayama 700-8530, Japan

**Keywords:** crystal structure, 4-chloro-2-nitro­benzoic acid, 4(1*H*)-quinolinone, 4-hy­droxy­quinoline, hydrogen bond, keto–enol tautomerization, Hirshfeld surface

## Abstract

The title compound, C_9_H_7.5_NO·C_7_H_3.5_ClNO_4_, was analysed as a disordered structure over two states, *viz*. co-crystal and salt, accompanied by a keto–enol tautomerization in the base mol­ecule. The co-crystal is 4-chloro-2-nitro­benzoic acid–quinolin-4(1*H*)-one (1/1), C_7_H_4_ClNO_4_·C_9_H_7_NO, and the salt is 4-hy­droxy­quinolinium 4-chloro-2-nitro­benzoate, C_9_H_8_NO^+^·C_7_H_3_ClNO_4_^−^. In the compound, the acid and base mol­ecules are held together by a short hydrogen bond [O⋯O = 2.4393 (15) Å], in which the H atom is disordered over two positions with equal occupancies. In the crystal, the hydrogen-bonded acid–base units are linked by N—H⋯O and C—H⋯O hydrogen bonds, forming a tape structure along the *a-*axis direction. The tapes are stacked into a layer parallel to the *ab* plane *via* π–π inter­actions [centroid–centroid distances = 3.5504 (8)–3.9010 (11) Å]. The layers are further linked by another C—H⋯O hydrogen bond, forming a three-dimensional network. Hirshfeld surfaces for the title compound mapped over shape-index and *d*_norm_ were generated to visualize the inter­molecular inter­actions.

## Abstract 2

The title compound was analysed as a disordered structure over two states, *viz*. co-crystal and salt, accompanied by a keto–enol tautomerization in the base mol­ecule. In the compound, the acid and base mol­ecules are linked by a short hydrogen bond [O⋯O = 2.4393 (15) Å], in which the H atom is disordered over two positions with equal occupancies.

## Chemical context   

In our previous study on *D*—H⋯*A* hydrogen bonding (*D* = N, O, or C, *A* = N, O or Cl) in chloro- and nitro-substituted benzoic acid–pyridine derivative systems, we have shown that several compounds, namely, three compounds of quinoline with 3-chloro-2-nitro­benzoic acid, 4-chloro-2-nitro­benzoic acid and 5-chloro-2-nitro­benzoic acid (Gotoh & Ishida, 2009 ▸), two compounds of phthalazine with 3-chloro-2-nitro­benzoic acid and 4-chloro-2-nitorbenzoic acid (Gotoh & Ishida, 2011 ▸), and 3-chloro-2-nitro­benzoic acid–iso­quinoline (Gotoh & Ishida, 2015 ▸), have a short double-well O⋯H⋯N hydrogen bond between the carb­oxy O atom and the aromatic N atom. Hy­droxy­quinolines, which have hydrogen-bond acceptor as well as donor groups, appear attractive as a base mol­ecule in the above systems for investigating the hydrogen bonds (Babu & Chandrasekaran, 2014 ▸; Gotoh & Ishida, 2019 ▸). We report here the crystal structure of the title compound, in which there exists another type of short double-well hydrogen bond, namely, an O⋯H⋯O hydrogen bond between the acid and base mol­ecules, accompanied by a keto–enol tautomerization of the base mol­ecule.
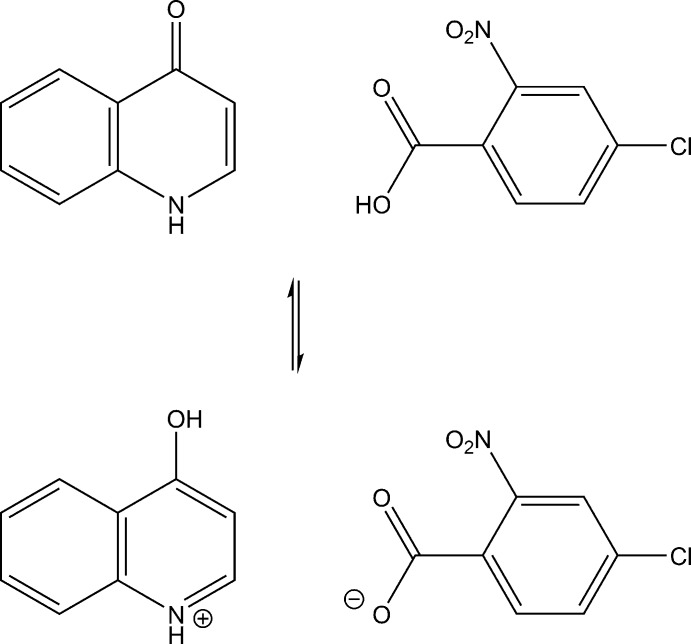


## Structural commentary   

**Figure 1:**
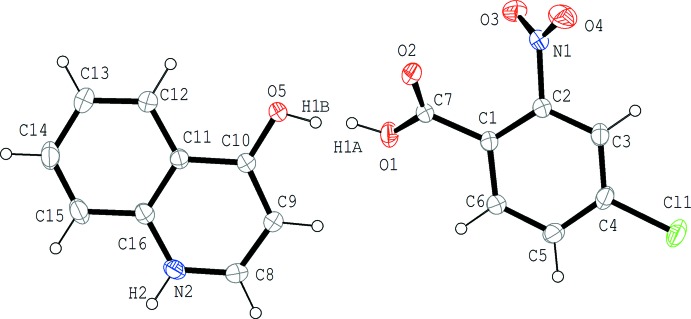
The mol­ecular structure of the title compound, showing the atom-numbering scheme. Displacement ellipsoids are drawn at the 50% probability level and H atoms are shown as small spheres of arbitrary radii.

**Figure 2:**
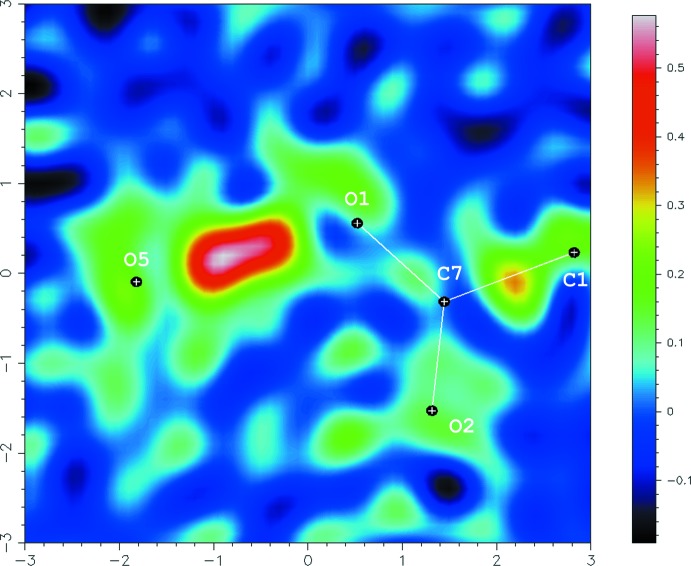
A difference-Fourier map of the title compound associated with the O⋯H⋯O hydrogen bond between the acid and the base. The map was calculated on the plane of atoms O1, C7 and O5 from a model containing all atoms apart from the H atom in the hydrogen bond.

The mol­ecular structure of the title compound is shown in Fig. 1 ▸. The acid and base mol­ecules are held together by a short hydrogen bond between atom O1 of the acid mol­ecule and atom O5 of the base [O1⋯O5 = 2.4393 (15) Å; Table 1 ▸]. In the hydrogen bond, the H atom is disordered as indicated in the difference-Fourier map (Fig. 2 ▸), in which a broad peak along the line connecting the two O atoms is observed. Although two distinct peaks were not clearly observed in the map, the H atom was successfully analysed as being disordered over two positions of the O1 and O5 sites with equal occupancies. The title compound is, thus, inter­preted as a disordered structure over two states, *viz.* the co-crystal, 4-chloro-2-nitro­benzoic acid–4(1*H*)-quinolinone (1/1), and the salt, 4-hy­droxy­quinolinium 4-chloro-2-nitro­benzoate, accompanied by a keto–enol tautomerization in the base mol­ecule. The C10—O5 bond length [1.2956 (18) Å] is inter­mediate between a C—O single bond [1.36 Å in phenol] and a C=O double bond [1.23 Å in ketones of the (C_ar_)_2_—C=O type] (Allen *et al.*, 1987 ▸), supporting that hypothesis that the base mol­ecule has an inter­mediate state between the keto and enol forms.

In the hydrogen-bonded acid–base unit, the benzene ring (C1–C6) of the acid mol­ecule and the quinoline ring system (N2/C8–C16) of the base are slightly inclined to each other by a dihedral angle of 10.27 (6)°, while the carb­oxy group (O1/C7/O2) is twisted by 38.66 (18) and 45.93 (18)°, respectively, with respect to the C1–C6 ring and the N2/C8–C16 ring system. The dihedral angle between the C1–C6 ring and the nitro group (O3/N1/O4) is 50.33 (19)°.

## Supra­molecular features   

**Figure 3:**
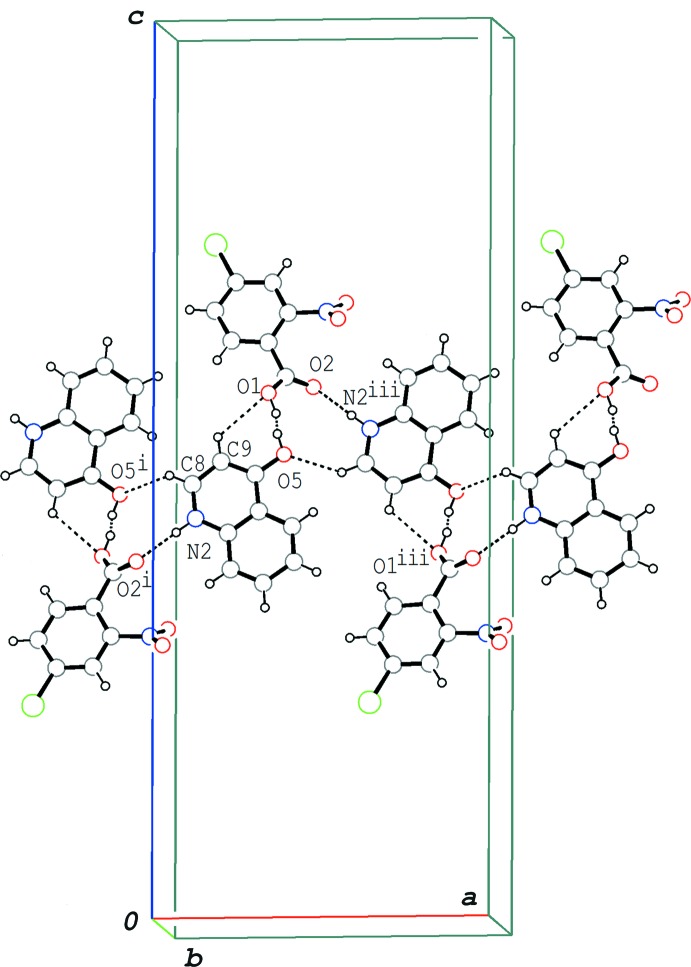
A packing diagram of the title compound, showing the hydrogen-bonded tape structure formed *via* the O⋯H⋯O, N—H⋯O and C—H⋯O hydrogen bonds (dashed lines). [Symmetry codes: (i) *x* − 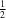, −*y* + 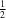, −*z* + 1; (iii) *x* + 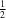, −*y* + 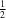, −*z* + 1.]

**Figure 4:**
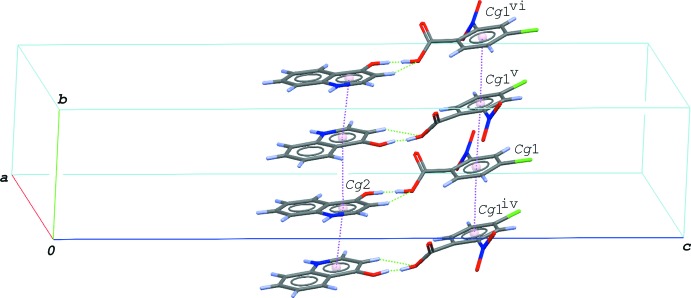
A packing diagram of the title compound, showing hydrogen-bonded acid-base units stacked along the *b* axis *via* the π–π inter­actions (magenta dashed lines). The π–π inter­actions including the centroid of the C11–C16 ring of the base (*Cg*3) are omitted for clarity. The O⋯H⋯O and C—H⋯O hydrogen bonds are indicated by green dashed lines. [Symmetry codes: (iv) −*x* + 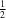, *y* − 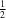, *z*; (v) −*x* + 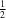, *y* + 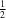, *z*; (vi) *x*, *y* + 1, *z*.]

In the crystal of the title compound, the hydrogen-bonded acid–base units are linked by N—H⋯O and C—H⋯O hydrogen bonds (N2—H2⋯O2^i^, C8—H8⋯O5^i^ and C9—H9⋯O1^i^; symmetry code as in Table 1 ▸), forming a tape structure along the *a* axis (Fig. 3 ▸). The tapes are stacked into a layer parallel to the *ab* plane *via* π–π inter­actions formed between the acid mol­ecules and between the base mol­ecules (Fig. 4 ▸); the centroid–centroid distances are 3.5504 (8), 3.7141 (9), 3.7382 (10) and 3.9010 (11) Å, respectively, for *Cg*1⋯*Cg*1^iv^, *Cg*2⋯*Cg*2^iv^, *Cg*3⋯*Cg*2^iv^ and *Cg*3⋯*Cg*3^iv^, where *Cg*1, *Cg*2 and *Cg*3 are the centroids of the C1–C6 ring of the acid mol­ecule, and the N2/C8–C11/C16 and C11–C16 rings of the base mol­ecule, respectively [symmetry code: (iv) −*x* + 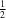, *y* − 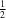, *z*]. The layers are further linked by another C—H⋯O hydrogen bond (C3—H3⋯O4^ii^; Table 1 ▸), forming a three-dimensional network.

**Figure 5:**
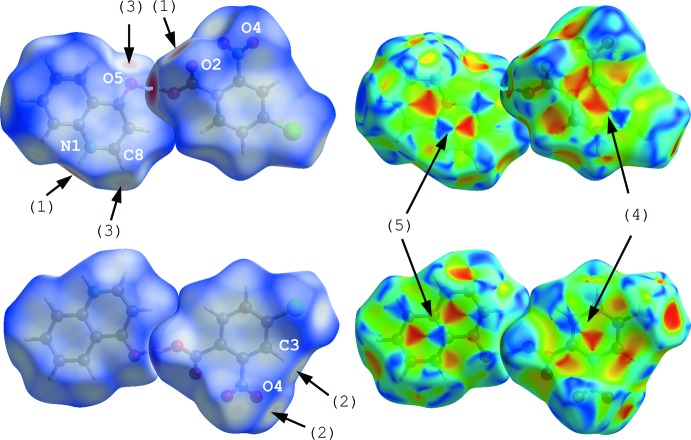
Hirshfeld surfaces (front and back views) for the title compound mapped over *d*_norm_ and shape-index, indicating the N—H⋯O [arrows (1)], C—H⋯O [arrows (2) and (3)] and π–π [arrows (4) and (5)] inter­actions.

In order to visualize the inter­molecular inter­actions, Hirshfeld surfaces for the acid and base mol­ecules of the title compound, mapped over shape-index and *d*_norm_ (Turner *et al.*, 2017 ▸; McKinnon *et al.*, 2004 ▸, 2007 ▸), were generated (Fig. 5 ▸). Inter­molecular hydrogen bonds of N2—H2⋯O2^i^, C3—H3⋯O4^ii^ and C8—H8⋯O5^i^ (Table 1 ▸) are represented as faint-red spots on the *d*_norm_ surfaces [arrows (1)–(3)]. The π–π inter­actions between the benzene rings of the acid mol­ecules [*Cg*1⋯*Cg*1^iv^] and between the quinoline ring systems of the base mol­ecules [*Cg*2⋯*Cg*2^iv^, *Cg*3⋯*Cg*2^iv^ and *Cg*3⋯*Cg*3^iv^; symmetry code: (iv) −*x* + 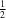, *y* − 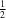, *z*] are indicated by blue and red triangles on the shape-index surfaces [arrows (4) and (5)].

## Database survey   

A search of the Cambridge Structural Database (Version 5.40, last update August 2019; Groom *et al.*, 2016 ▸) for organic co-crystals/salts of 4(1*H*)-quinolinone (keto tautomer) showed one structure, namely, 4-amino-1-(2-(hy­droxy­meth­yl)-1,3-oxa­thio­lan-5-yl)-2(1*H*)-pyrimidinone 4(1*H*)-quinolinone (refcode COWTAK; Bhatt *et al.*, 2009 ▸). The structure of the 4(1*H*)-quinolinone itself was reported by Nasiri *et al.* (2006 ▸; NICIOZ). The C=O bond length in COWTAK is 1.265 (7) Å and those in NICIOZ are 1.2686 (16) and 1.2742 (15) Å, which are shorter than the C10—O5 bond length of 1.2956 (18) Å in the title compound. No structure was found in the CSD for organic co-crystals/salts of 4-hy­droxy­quinoline (enol tautomer). A search for organic co-crystals/salts of 4-chloro-2-nitro­benzoic acid with base mol­ecules gave eight compounds. Of these compounds, disorder of H atom between the acid O atom and the base N atom was observed in two compounds of 4-chloro-2-nitro­benzoic acid with quinoline (AJIWUM; Gotoh & Ishida, 2009 ▸) and phthalazine (CALKAD; Gotoh & Ishida, 2011 ▸).

## Synthesis and crystallization   

Single crystals of the title compound suitable for X-ray diffraction analysis were obtained by slow evaporation from an aceto­nitrile solution (130 ml) of 4-hy­droxy­quinoline (0.075 g) with 4-chloro-2-nitro­benzoic acid (0.106 g) in a 1:1 molar ratio at room temperature.

## Refinement   

Crystal data, data collection and structure refinement details are summarized in Table 2 ▸. All H atoms except one H atom between the two O atoms (O1 and O5) of the acid and base mol­ecules were found in a difference-Fourier map. A broad peak in a difference-Fourier map between atoms O1 and O5 was observed (Fig. 2 ▸). Although two distinct peaks were not observed in the map, the H atom between the O atoms was analysed using a model of an H atom disordered over two positions. The occupancies of the two sites were refined to 0.47 (4) and 0.53 (4) for H1*A* (O1 site) and H1*B* (O5 site), respectively, with bond restraints of O—H = 0.84 (1) Å and with *U*_iso_(H) = 1.5*U*_eq_(O). In the final refinement, the occupancies were fixed at 0.5, and one outlier (6,8,13) was omitted. The N-bound H atom was refined freely [refined distance: N2—H2 = 0.89 (2) Å]. Other H atoms were positioned geometrically (C—H = 0.95 Å) and treated as riding, with *U*_iso_(H) = 1.2*U*_eq_(C).

## Supplementary Material

Crystal structure: contains datablock(s) global, I. DOI: 10.1107/S205698901901497X/lh5935sup1.cif

Structure factors: contains datablock(s) I. DOI: 10.1107/S205698901901497X/lh5935Isup2.hkl

CCDC references: 1963942, 1963942

Additional supporting information:  crystallographic information; 3D view; checkCIF report

## Figures and Tables

**Table 1:** Hydrogen-bond geometry (Å, °)

*D*—H⋯*A*	*D*—H	H⋯*A*	*D*⋯*A*	*D*—H⋯*A*
O1—H1*A*⋯O5	0.84 (3)	1.61 (2)	2.4393 (15)	172 (5)
O5—H1*B*⋯O1	0.84 (2)	1.60 (2)	2.4393 (15)	173 (4)
N2—H2⋯O2^i^	0.89 (2)	1.86 (2)	2.7475 (18)	176 (2)
C3—H3⋯O4^ii^	0.95	2.53	3.469 (2)	170
C8—H8⋯O5^i^	0.95	2.45	3.208 (2)	137
C9—H9⋯O1	0.95	2.51	3.121 (2)	123

Symmetry codes: (i) 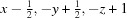; (ii) 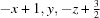.

**Table 2:** Experimental details

Crystal data	
Chemical formula	C_7_H_3.5_ClNO_4_·C_9_H_7.5_NO
*M*_r_	346.73
Crystal system, space group	Orthorhombic, *P* *b* *c* *n*
Temperature (K)	190
*a*, *b*, *c* (Å)	12.6336 (8), 7.0701 (3), 33.5956 (15)
*V* (Å^3^)	3000.8 (3)
*Z*	8
Radiation type	Mo *K*α
μ (mm^−1^)	0.29
Crystal size (mm)	0.35 × 0.28 × 0.09

Data collection	
Diffractometer	Rigaku R-AXIS RAPIDII
Absorption correction	Numerical (*NUMABS*; Higashi, 1999 ▸)
*T*_min_, *T*_max_	0.939, 0.975
No. of measured, independent and observed [*I* > 2σ(*I*)] reflections	36709, 4380, 3235
*R*_int_	0.052
(sin θ/λ)_max_ (Å^−1^)	0.704

Refinement	
*R*[*F* ^2^ > 2σ(*F* ^2^)], *wR*(*F* ^2^), *S*	0.046, 0.136, 1.13
No. of reflections	4380
No. of parameters	227
No. of restraints	2
H-atom treatment	H atoms treated by a mixture of independent and constrained refinement
Δρ_max_, Δρ_min_ (e Å^−3^)	0.39, −0.42

Computer programs: *PROCESS-AUTO* (Rigaku, 2006 ▸), *SHELXT2018* (Sheldrick, 2015*a* ▸), *SHELXL2018* (Sheldrick, 2015*b* ▸), *ORTEP-3 for Windows* and *WinGX* (Farrugia, 2012 ▸); *Mercury* (Macrae *et al.*, 2008 ▸), *CrystalStructure* (Rigaku, 2018 ▸) and *PLATON* (Spek, 2015 ▸).

## supplementary crystallographic information

### Crystal data

**Table:**

C_7_H_3.5_ClNO_4_·C_9_H_7.5_NO	*D*_x_ = 1.535 Mg m^−^^3^
*M**_r_* = 346.73	Mo *K*α radiation, λ = 0.71075 Å
Orthorhombic, *P**b**c**n*	Cell parameters from 23445 reflections
*a* = 12.6336 (8) Å	θ = 3.0–30.2°
*b* = 7.0701 (3) Å	µ = 0.28 mm^−^^1^
*c* = 33.5956 (15) Å	*T* = 190 K
*V* = 3000.8 (3) Å^3^	Platelet, colorless
*Z* = 8	0.35 × 0.28 × 0.09 mm
*F*(000) = 1424.00	

### Data collection

**Table:**

Rigaku R-AXIS RAPIDII diffractometer	3235 reflections with *I* > 2σ(*I*)
Detector resolution: 10.000 pixels mm^-1^	*R*_int_ = 0.052
ω scans	θ_max_ = 30.0°, θ_min_ = 3.2°
Absorption correction: numerical (*NUMABS*; Higashi, 1999)	*h* = −17→17
*T*_min_ = 0.939, *T*_max_ = 0.975	*k* = −9→9
36709 measured reflections	*l* = −47→47
4380 independent reflections	

### Refinement

**Table:**

Refinement on *F*^2^	Primary atom site location: structure-invariant direct methods
Least-squares matrix: full	Secondary atom site location: difference Fourier map
*R*[*F*^2^ > 2σ(*F*^2^)] = 0.046	Hydrogen site location: mixed
*wR*(*F*^2^) = 0.136	H atoms treated by a mixture of independent and constrained refinement
*S* = 1.13	*w* = 1/[σ^2^(*F*_o_^2^) + (0.0641*P*)^2^ + 0.6358*P*] where *P* = (*F*_o_^2^ + 2*F*_c_^2^)/3
4380 reflections	(Δ/σ)_max_ = 0.002
227 parameters	Δρ_max_ = 0.39 e Å^−^^3^
2 restraints	Δρ_min_ = −0.42 e Å^−^^3^

### Special details

**Table:**

Geometry. All esds (except the esd in the dihedral angle between two l.s. planes) are estimated using the full covariance matrix. The cell esds are taken into account individually in the estimation of esds in distances, angles and torsion angles; correlations between esds in cell parameters are only used when they are defined by crystal symmetry. An approximate (isotropic) treatment of cell esds is used for estimating esds involving l.s. planes.

### Fractional atomic coordinates and isotropic or equivalent isotropic displacement parameters (Å^2^)

**Table:**

	*x*	*y*	*z*	*U*_iso_*/*U*_eq_	Occ. (<1)
Cl1	0.14687 (4)	0.53104 (8)	0.76188 (2)	0.05607 (17)	
O1	0.32791 (10)	0.20128 (19)	0.58896 (3)	0.0439 (3)	
H1A	0.351 (4)	0.185 (8)	0.5658 (6)	0.066*	0.5
O2	0.44512 (9)	0.43255 (18)	0.60030 (3)	0.0423 (3)	
O3	0.52878 (10)	0.26978 (19)	0.67248 (4)	0.0497 (3)	
O4	0.53263 (11)	0.5533 (2)	0.69656 (5)	0.0636 (4)	
O5	0.37717 (9)	0.15689 (18)	0.51938 (3)	0.0376 (3)	
H1B	0.356 (3)	0.177 (7)	0.5427 (6)	0.056*	0.5
N1	0.48587 (11)	0.4153 (2)	0.68364 (4)	0.0404 (3)	
N2	0.11801 (11)	0.1179 (2)	0.44816 (4)	0.0379 (3)	
C1	0.31559 (12)	0.3666 (2)	0.64897 (4)	0.0318 (3)	
C2	0.37014 (12)	0.4213 (2)	0.68293 (4)	0.0333 (3)	
C3	0.32058 (13)	0.4729 (2)	0.71785 (5)	0.0373 (3)	
H3	0.360145	0.511101	0.740510	0.045*	
C4	0.21176 (14)	0.4671 (2)	0.71868 (5)	0.0377 (4)	
C5	0.15375 (13)	0.4103 (2)	0.68590 (5)	0.0389 (4)	
H5	0.078678	0.405323	0.687096	0.047*	
C6	0.20643 (12)	0.3610 (2)	0.65135 (4)	0.0345 (3)	
H6	0.166717	0.322352	0.628754	0.041*	
C7	0.36954 (12)	0.3319 (2)	0.60966 (4)	0.0334 (3)	
C8	0.10616 (13)	0.1492 (2)	0.48692 (5)	0.0381 (3)	
H8	0.036732	0.163593	0.497354	0.046*	
C9	0.19038 (13)	0.1612 (2)	0.51224 (5)	0.0357 (3)	
H9	0.179065	0.181221	0.539873	0.043*	
C10	0.29314 (11)	0.1441 (2)	0.49744 (4)	0.0306 (3)	
C11	0.30605 (12)	0.1122 (2)	0.45544 (4)	0.0306 (3)	
C12	0.40628 (13)	0.0928 (2)	0.43769 (5)	0.0385 (4)	
H12	0.468363	0.099954	0.453575	0.046*	
C13	0.41438 (16)	0.0636 (3)	0.39760 (5)	0.0492 (4)	
H13	0.482176	0.050157	0.385705	0.059*	
C14	0.32353 (17)	0.0534 (3)	0.37403 (5)	0.0533 (5)	
H14	0.330455	0.035088	0.346139	0.064*	
C15	0.22572 (16)	0.0693 (3)	0.39020 (5)	0.0459 (4)	
H15	0.164531	0.059820	0.373885	0.055*	
C16	0.21571 (12)	0.0997 (2)	0.43129 (4)	0.0331 (3)	
H2	0.0611 (18)	0.107 (3)	0.4325 (6)	0.052 (6)*	

### Atomic displacement parameters (Å^2^)

**Table:**

	*U*^11^	*U*^22^	*U*^33^	*U*^12^	*U*^13^	*U*^23^
Cl1	0.0601 (3)	0.0749 (3)	0.0332 (2)	0.0104 (2)	0.01812 (19)	0.00122 (19)
O1	0.0488 (7)	0.0572 (7)	0.0257 (5)	−0.0068 (6)	0.0010 (5)	−0.0078 (5)
O2	0.0363 (6)	0.0585 (7)	0.0321 (5)	−0.0033 (5)	0.0060 (5)	−0.0043 (5)
O3	0.0385 (7)	0.0637 (8)	0.0468 (7)	0.0164 (6)	0.0004 (5)	0.0024 (6)
O4	0.0434 (8)	0.0792 (10)	0.0681 (9)	−0.0088 (7)	−0.0108 (7)	−0.0217 (8)
O5	0.0290 (5)	0.0565 (7)	0.0273 (5)	0.0005 (5)	−0.0025 (4)	−0.0066 (5)
N1	0.0338 (7)	0.0594 (8)	0.0282 (6)	0.0038 (6)	−0.0041 (5)	−0.0013 (6)
N2	0.0314 (7)	0.0430 (7)	0.0391 (7)	0.0016 (6)	−0.0090 (6)	−0.0023 (6)
C1	0.0325 (7)	0.0384 (8)	0.0245 (6)	0.0037 (6)	−0.0003 (5)	0.0010 (6)
C2	0.0313 (7)	0.0415 (8)	0.0271 (7)	0.0038 (6)	−0.0007 (5)	0.0011 (6)
C3	0.0424 (9)	0.0437 (8)	0.0257 (7)	0.0029 (7)	0.0007 (6)	−0.0008 (6)
C4	0.0429 (9)	0.0421 (8)	0.0281 (7)	0.0061 (7)	0.0093 (6)	0.0020 (6)
C5	0.0317 (8)	0.0484 (9)	0.0366 (8)	0.0036 (7)	0.0045 (6)	0.0062 (7)
C6	0.0315 (8)	0.0424 (8)	0.0297 (7)	0.0019 (6)	−0.0004 (6)	0.0028 (6)
C7	0.0307 (7)	0.0447 (8)	0.0247 (6)	0.0055 (6)	−0.0020 (5)	0.0011 (6)
C8	0.0294 (8)	0.0433 (8)	0.0417 (8)	0.0011 (6)	0.0014 (6)	0.0002 (7)
C9	0.0323 (8)	0.0443 (8)	0.0305 (7)	0.0008 (6)	0.0027 (6)	−0.0010 (6)
C10	0.0298 (7)	0.0337 (7)	0.0282 (7)	−0.0004 (6)	−0.0010 (5)	−0.0004 (6)
C11	0.0321 (7)	0.0320 (7)	0.0277 (7)	0.0004 (6)	−0.0008 (5)	−0.0002 (6)
C12	0.0345 (8)	0.0485 (9)	0.0326 (7)	0.0000 (7)	0.0011 (6)	−0.0042 (7)
C13	0.0492 (10)	0.0633 (11)	0.0350 (8)	0.0040 (9)	0.0094 (7)	−0.0065 (8)
C14	0.0653 (13)	0.0669 (13)	0.0279 (8)	0.0087 (10)	0.0002 (8)	−0.0072 (8)
C15	0.0550 (11)	0.0523 (10)	0.0304 (7)	0.0077 (8)	−0.0124 (7)	−0.0048 (7)
C16	0.0365 (8)	0.0318 (7)	0.0311 (7)	0.0018 (6)	−0.0042 (6)	−0.0016 (6)

### Geometric parameters (Å, º)

**Table:**

Cl1—C4	1.7271 (16)	C5—C6	1.383 (2)
O1—C7	1.270 (2)	C5—H5	0.9500
O1—H1A	0.841 (10)	C6—H6	0.9500
O2—C7	1.2317 (19)	C8—C9	1.365 (2)
O3—N1	1.2218 (19)	C8—H8	0.9500
O4—N1	1.220 (2)	C9—C10	1.395 (2)
O5—C10	1.2956 (18)	C9—H9	0.9500
O5—H1B	0.841 (10)	C10—C11	1.438 (2)
N1—C2	1.463 (2)	C11—C16	1.403 (2)
N2—C8	1.329 (2)	C11—C12	1.406 (2)
N2—C16	1.364 (2)	C12—C13	1.366 (2)
N2—H2	0.90 (2)	C12—H12	0.9500
C1—C6	1.382 (2)	C13—C14	1.396 (3)
C1—C2	1.388 (2)	C13—H13	0.9500
C1—C7	1.506 (2)	C14—C15	1.355 (3)
C2—C3	1.379 (2)	C14—H14	0.9500
C3—C4	1.376 (2)	C15—C16	1.403 (2)
C3—H3	0.9500	C15—H15	0.9500
C4—C5	1.382 (2)		
			
C7—O1—H1A	117 (4)	O1—C7—C1	114.27 (14)
C10—O5—H1B	106 (3)	N2—C8—C9	122.21 (15)
O4—N1—O3	124.57 (15)	N2—C8—H8	118.9
O4—N1—C2	117.80 (14)	C9—C8—H8	118.9
O3—N1—C2	117.58 (14)	C8—C9—C10	119.87 (14)
C8—N2—C16	121.64 (14)	C8—C9—H9	120.1
C8—N2—H2	120.1 (14)	C10—C9—H9	120.1
C16—N2—H2	118.3 (14)	O5—C10—C9	123.62 (14)
C6—C1—C2	117.13 (14)	O5—C10—C11	118.44 (13)
C6—C1—C7	119.84 (13)	C9—C10—C11	117.94 (13)
C2—C1—C7	122.78 (14)	C16—C11—C12	118.77 (14)
C3—C2—C1	123.20 (15)	C16—C11—C10	119.00 (13)
C3—C2—N1	116.59 (14)	C12—C11—C10	122.23 (13)
C1—C2—N1	120.11 (13)	C13—C12—C11	120.00 (16)
C4—C3—C2	117.59 (15)	C13—C12—H12	120.0
C4—C3—H3	121.2	C11—C12—H12	120.0
C2—C3—H3	121.2	C12—C13—C14	120.35 (17)
C3—C4—C5	121.49 (15)	C12—C13—H13	119.8
C3—C4—Cl1	118.92 (13)	C14—C13—H13	119.8
C5—C4—Cl1	119.59 (13)	C15—C14—C13	121.21 (16)
C4—C5—C6	119.13 (15)	C15—C14—H14	119.4
C4—C5—H5	120.4	C13—C14—H14	119.4
C6—C5—H5	120.4	C14—C15—C16	119.31 (16)
C1—C6—C5	121.44 (15)	C14—C15—H15	120.3
C1—C6—H6	119.3	C16—C15—H15	120.3
C5—C6—H6	119.3	N2—C16—C15	120.32 (15)
O2—C7—O1	126.98 (14)	N2—C16—C11	119.34 (14)
O2—C7—C1	118.71 (14)	C15—C16—C11	120.34 (15)
			
C6—C1—C2—C3	−1.4 (2)	C16—N2—C8—C9	−1.3 (3)
C7—C1—C2—C3	172.84 (15)	N2—C8—C9—C10	1.2 (3)
C6—C1—C2—N1	174.88 (14)	C8—C9—C10—O5	178.85 (15)
C7—C1—C2—N1	−10.9 (2)	C8—C9—C10—C11	−0.5 (2)
O4—N1—C2—C3	−50.5 (2)	O5—C10—C11—C16	−179.45 (14)
O3—N1—C2—C3	127.10 (16)	C9—C10—C11—C16	0.0 (2)
O4—N1—C2—C1	132.98 (17)	O5—C10—C11—C12	0.7 (2)
O3—N1—C2—C1	−49.4 (2)	C9—C10—C11—C12	−179.91 (15)
C1—C2—C3—C4	0.7 (2)	C16—C11—C12—C13	0.4 (2)
N1—C2—C3—C4	−175.67 (14)	C10—C11—C12—C13	−179.68 (16)
C2—C3—C4—C5	0.5 (2)	C11—C12—C13—C14	0.2 (3)
C2—C3—C4—Cl1	−179.91 (13)	C12—C13—C14—C15	−1.0 (3)
C3—C4—C5—C6	−0.9 (3)	C13—C14—C15—C16	1.1 (3)
Cl1—C4—C5—C6	179.46 (13)	C8—N2—C16—C15	−179.16 (16)
C2—C1—C6—C5	0.9 (2)	C8—N2—C16—C11	0.7 (2)
C7—C1—C6—C5	−173.51 (15)	C14—C15—C16—N2	179.37 (17)
C4—C5—C6—C1	0.2 (3)	C14—C15—C16—C11	−0.5 (3)
C6—C1—C7—O2	138.07 (16)	C12—C11—C16—N2	179.85 (14)
C2—C1—C7—O2	−36.0 (2)	C10—C11—C16—N2	0.0 (2)
C6—C1—C7—O1	−39.9 (2)	C12—C11—C16—C15	−0.3 (2)
C2—C1—C7—O1	146.07 (15)	C10—C11—C16—C15	179.82 (15)

### Hydrogen-bond geometry (Å, º)

**Table:**

*D*—H···*A*	*D*—H	H···*A*	*D*···*A*	*D*—H···*A*
O1—H1*A*···O5	0.84 (3)	1.61 (2)	2.4393 (15)	172 (5)
O5—H1*B*···O1	0.84 (2)	1.60 (2)	2.4393 (15)	173 (4)
N2—H2···O2^i^	0.89 (2)	1.86 (2)	2.7475 (18)	176 (2)
C3—H3···O4^ii^	0.95	2.53	3.469 (2)	170
C8—H8···O5^i^	0.95	2.45	3.208 (2)	137
C9—H9···O1	0.95	2.51	3.121 (2)	123

Symmetry codes: (i) *x*−1/2, −*y*+1/2, −*z*+1; (ii) −*x*+1, *y*, −*z*+3/2.
